# RPA-CRISPR/Cas12a-Mediated Isothermal Amplification Technology for Visual Detection of *Fusarium proliferatum*

**DOI:** 10.3390/plants15152352

**Published:** 2026-07-30

**Authors:** Bingyan Zheng, Chenyun Guan, Jiahui Zang, Xiaoqiao Xu, Chun Yang, Xiaorui Zhang, Tingting Dai

**Affiliations:** Co-Innovation Center for the Sustainable Forestry in Southern China, Nanjing Forestry University, Nanjing 210037, China; 18260036961@139.com (B.Z.); guanchenyun@nifu.edu.cn (C.G.); zjh20011009@163.com (J.Z.); 15398228048@163.com (X.X.); yangchun0724@163.com (C.Y.); 17761709897@163.com (X.Z.)

**Keywords:** *Fusarium proliferatum*, recombinase polymerase amplification, CRISPR/Cas12a, rapid detection, molecular diagnosis

## Abstract

*Fusarium proliferatum* is a fungal pathogen with an exceptionally broad host range, infecting ornamental plants and forest trees such as *Populus* and *Cedrus deodara*, and causing heart rot, root rot, and other diseases that lead to severe yield losses. It can also produce mycotoxins, including fumonisins, which threaten human and animal health. Conventional detection relies on isolation, culture, and morphological identification, which are time-consuming and prone to misidentification. Here, we established a rapid, visual molecular detection method by combining recombinase polymerase amplification (RPA) with the CRISPR/Cas12a system, targeting the *F. proliferatum*-specific gene *Fpro_15953*. The assay exhibited high specificity: the *F. proliferatum* isolate from *C. deodara* tested positive, while 37 non-target isolates (10 other *Fusarium* species, 10 other fungi, 15 oomycetes, and 2 *Bursaphelenchus* nematodes) produced no detectable signal. Under isothermal conditions at 37 °C, after 10 min of RPA followed by 10 min of Cas12a cleavage, as little as 0.001 ng·μL^−1^ of *F. proliferatum* genomic DNA could be detected. The method further succeeded in detecting *F. proliferatum* in artificially inoculated *C. deodara* needles and *Populus* × *hopeiensis* stem and root segments. This RPA-CRISPR/Cas12a platform provides an efficient, accurate tool for *F. proliferatum* detection and supports rapid field diagnosis.

## 1. Introduction

*Fusarium proliferatum* is a phytopathogenic fungus with an exceptionally broad host range, first isolated and identified by Nirenberg in 1976 [1]. On forest trees, this species infects important coniferous and broadleaf species such as *Cedrus deodara* and *Populus* spp., causing leaf blight and shoot blight, which lead to extensive defoliation, shoot dieback, and severe reductions in ornamental and ecological value. It also infects major cereal crops including rice, maize, and sorghum, as well as economically important vegetables such as onion, garlic, and asparagus, and horticultural plants including mango, date palm, and *Cymbidium hybridum* [2,3,4,5,6]. In addition, the fungus can survive saprophytically in soil and on plant debris and may act as an opportunistic human pathogen, causing onychomycosis, endophthalmitis, and even disseminated infections [7,8]. Its ability to infect both plants and animals highlights its cross-kingdom pathogenic potential and raises concerns regarding the risk of transregional spread.

On *C. deodara* naturally infected in the field, we observed that *F. proliferatum* causes needle blight and shoot blight. Early symptoms begin as water-soaked or chlorotic small spots on needles, which gradually expand into reddish-brown to dark brown necrotic lesions. Under favorable conditions, the lesions coalesce, causing the entire needle of a branch to yellow, wither, and shed, eventually resulting in branch dieback. Under high humidity, sparse white to pale pink mycelial growth may be visible on the lesion surface. These symptoms closely resemble those caused by other foliar pathogens of *C. deodara* [9]; therefore, accurate identification of the causal species based solely on visual observation is extremely difficult, frequently leading to misdiagnosis and inappropriate disease control measures. On *Cedrus deodara*, in addition to the needle blight caused by *F. proliferatum*, similar symptoms such as needle necrosis and shoot dieback can also be induced by other foliar pathogens. On *Populus* × *hopeiensis*, canker and stem rot diseases caused by various *Fusarium* species and other fungi often exhibit overlapping symptoms, making field diagnosis based solely on visible signs highly unreliable. Therefore, a rapid and specific molecular tool is urgently needed to differentiate *F. proliferatum* from these confounding pathogens.

Traditional identification of *F. proliferatum* relies on isolation from the margin between diseased and healthy tissues, purification culture, morphological characterization (including macroconidia, microconidia, colony pigmentation, and growth rate) [2], and subsequent molecular confirmation through PCR amplification and sequencing of the internal transcribed spacer (ITS) region or translation elongation factor 1-α (*TEF1*) gene [10,11,12]. This entire procedure typically requires 5–7 days, along with a thermocycler, gel electrophoresis equipment, a sequencing platform, and trained personnel, making it unsuitable for early disease warning or on-site rapid detection. Although real-time quantitative PCR (qPCR) and nested PCR provide significantly higher sensitivity and specificity than conventional PCR [13], they still depend on expensive thermal cycling instruments and skilled operators, which limits their field applicability.

Isothermal amplification technologies, particularly loop-mediated isothermal amplification (LAMP) and recombinase polymerase amplification (RPA), have attracted considerable attention because they eliminate the need for thermal cycling [14,15]. However, LAMP requires complex primer design and is prone to non-specific amplification [14,16], whereas RPA, although faster and simpler, can generate false-positive results due to primer-dimers [15,17,18], and the result readout often relies on turbidity or fluorescent dyes, which is not sufficiently straightforward under field conditions [16,18]. These limitations have driven the search for more robust, instrument-free detection platforms.

The integration of CRISPR/Cas systems with isothermal amplification gave rise to the DNA endonuclease-targeted CRISPR trans reporter (DETECTR) system, first reported in 2018 [19]. The RPA-CRISPR/Cas12a assay is based on a simple principle: RPA isothermally amplifies the target DNA, after which the Cas12a-crRNA complex specifically recognizes the amplicon and activates its non-specific *trans*-cleavage activity, cleaving a single-stranded DNA reporter probe labeled with a fluorophore and a quencher at each end, thereby releasing a detectable fluorescence signal [19,20]. This platform offers the following advantages: (i) extremely high specificity, with Cas12a capable of discriminating single-nucleotide variants; (ii) ultra-high sensitivity, reaching sub-attomolar levels; (iii) rapid turnaround, typically completed within 30–60 min; and (iv) simple result interpretation, as the fluorescence can be observed directly with a portable blue light transilluminator or even by the naked eye [19,21,22].

In recent years, RPA-CRISPR/Cas12a-based methods have been widely applied for the detection of diverse pathogens, including viruses (e.g., African swine fever virus, SARS-CoV-2) [23,24], bacteria (e.g., *Mycobacterium tuberculosis*) [25], and plant pathogenic fungi such as *Puccinia striiformis* f. sp. *tritici* [26], *Fusarium graminearum* [27], *Botrytis cinerea* [28], and *Fusarium oxysporum* [29].

**Figure 1:**
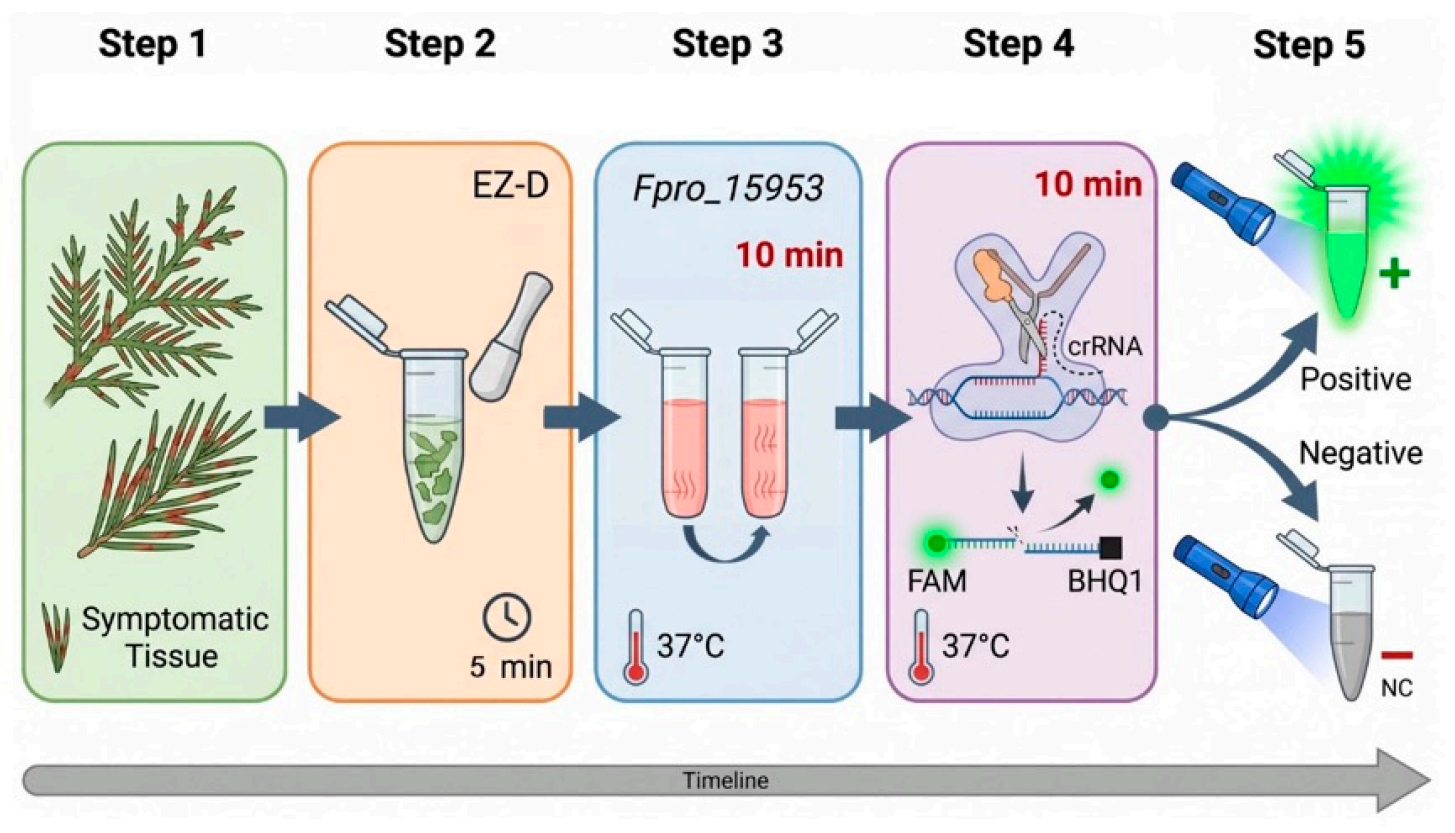
Workflow of RPA-CRISPR/Cas12a detection of *Fusarium proliferatum*. After sample collection, DNA is rapidly extracted by EZ-D, followed by RPA isothermal amplification (37 °C, 10 min). The amplification product is transferred to the CRISPR/Cas12a detection system (37 °C, 10 min). The result is interpreted by observing green fluorescence under a blue light LED transilluminator. NC: no-template control.

However, an RPA-CRISPR/Cas12a detection system specifically targeting *F. proliferatum* has not been reported to date [30]. Therefore, in this study, we aimed to develop a rapid, visual RPA-CRISPR/Cas12a assay for *F. proliferatum* by targeting the species-specific gene *Fpro_15953*. This method integrates crude DNA extraction, RPA amplification, and Cas12a cleavage into a streamlined workflow that can be completed in approximately 25–40 min (Figure 1); results are directly visualized using a portable fluorescence transilluminator after the reaction. Specificity tests demonstrated that the assay exhibited no cross-reactivity with other *Fusarium* species (e.g., *F. graminearum*, *F. verticillioides*) or other common *Cedrus deodara* pathogens. The establishment of this detection platform provides critical technical support for the future development of field-deployable detection kits and contributes to the early warning and precision management of *C. deodara* blight as well as diseases on other ornamental and forest trees.

## 2. Results

### 2.1. EZ-D Rapid Nucleic Acid Extraction

**Figure 2:**
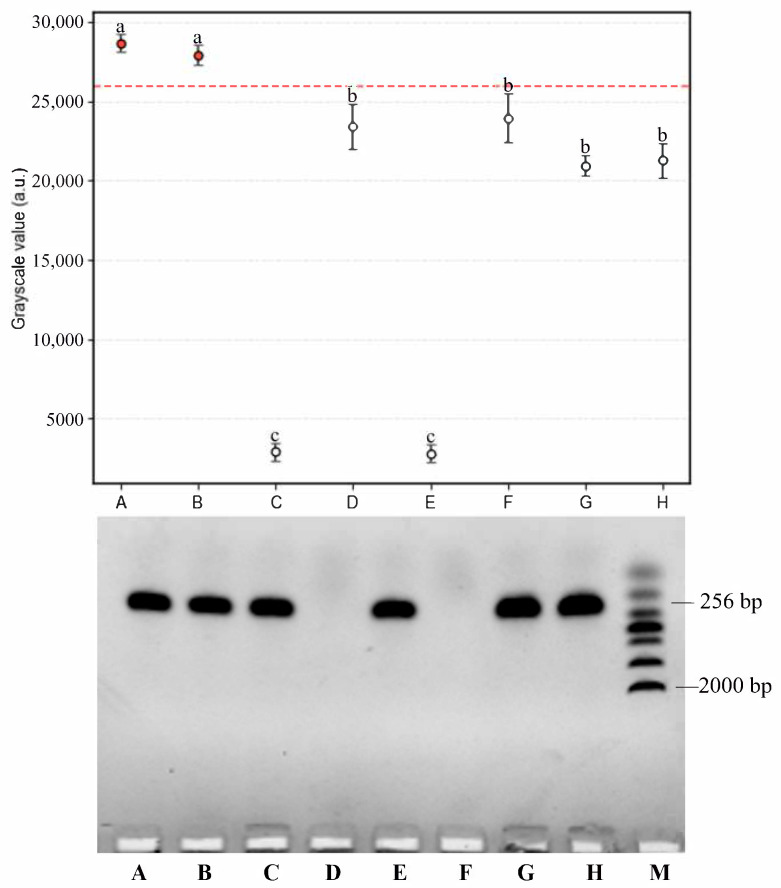
RPA amplification results of *Cedrus deodara* DNA extracted with different extraction buffers. Upper panel: Representative electrophoretogram. The target amplification fragment is 256 bp. M: DNA molecular weight marker (sizes labeled as 2000 bp and 256 bp); lanes (A–H) correspond to the 8 extraction buffers in Table 1. Lower panel: Scatter plot of gray values. Each dot represents one replicate, with horizontal lines representing mean ± standard deviation (SD). The red dashed line indicates the threshold (26,000 a.u.). One-way ANOVA showed that the fluorescence gray values of each extraction buffer were significantly different from the negative control. Different letters (a–c) indicate significant differences in gray values between groups (*p* < 0.05). Data represent the mean ± SD of three replicates (*n* = 3).

Using the EZ-D method described above, DNA was extracted from *Cedrus deodara* needles with each of the eight extraction solutions, and the supernatants were directly used for RPA amplification (target fragment approximately 256 bp). The amplification products were detected by 2% agarose gel electrophoresis, and the target bands were subjected to grayscale value analysis. The RPA electrophoresis results for each extraction solution are shown in Figure 2 upper panel, and the corresponding grayscale value quantitative analysis is presented as a scatter plot in the lower panel.From the electrophoretogram (Figure 2 upper panel), lanes A (TPS extraction buffer) and B (SDS extraction buffer) showed clear and bright bands, with the highest amplification efficiency; lanes D, F, G, and H showed visible but low-intensity bands; lanes C and E showed almost no specific bands. The gray value scatter plot (Figure 2 (lower panel)) further indicated that extraction buffer A had the highest gray value (28693.78 ± 468.43) and the best reproducibility; extraction buffer B was next; buffers C and E had very low gray values (<3000), presumably due to CTAB residue inhibiting the RPA reaction; buffers D and F had moderate gray values (approximately 23,000–24,000); buffers G and H had the lowest gray values (approximately 21,000). Comprehensive comparison showed that extraction buffer A provided the best quality DNA template and was most suitable for RPA amplification. Therefore, extraction buffer A (TPS: 100 mM Tris [pH 8.0], 1 M NaCl, 10 mM EDTA) was selected as the standard extraction buffer for the EZ-D method in this study.

**Table 1:** Formulations of 8 different extraction solutions for the EZ-D method.

Type	Extraction Solution	Composition
A	TPS extraction solution	100 mM Tris [pH 8.0], 1 M NaCl, 10 mM EDTA
B	SDS extraction solution	20 mM Tris [pH 8.0], 25 mM NaCl, 2.5 mM EDTA, 0.05% SDS
C	CTAB extraction solution	2% CTAB, 50 mM Tris [pH 8.0], 0.7 M NaCl, 10 mM EDTA
D	TPS/SDS extraction solution	0.005% SDS, 100 mM Tris [pH 8.0], 1 M NaCl, 10 mM EDTA
E	TPS/CTAB extraction solution	2% CTAB, 100 mM Tris [pH 8.0], 1 M NaCl, 10 mM EDTA
F	0.1 M NaOH extraction solution	0.1 M NaOH
G	Optimized NaOH extraction solution	0.1 M NaOH, 0.05% SDS, 2% PVP
H	Tween-20 extraction solution	50 mM Tris [pH 8.0], 150 mM NaCl, 2% PVP, 1% Tween-20

### 2.2. Optimization of the RPA-CRISPR/Cas12a Detection Method for Fusarium proliferatum

**Figure 3:**
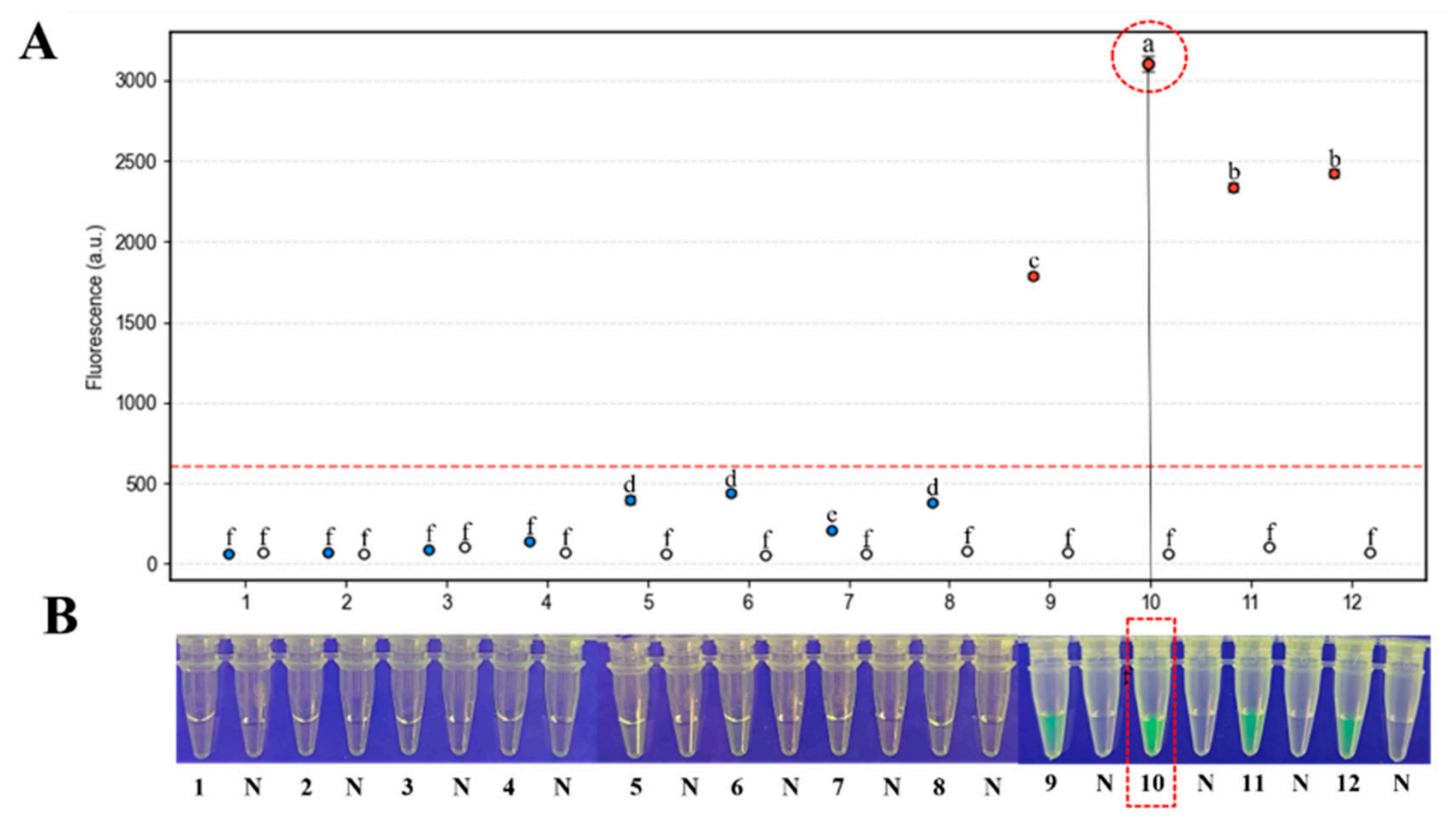
Optimization of crRNA and ssDNA reporter probe concentrations in the RPA-CRISPR/Cas12a detection system. Twelve different concentration combinations were tested: 1. 40 nM crRNA + 40 nM ssDNA reporter; 2. 80 nM crRNA + 500 nM ssDNA reporter; 3. 300 nM crRNA + 1.4 nM ssDNA reporter; 4. 0.5 µM crRNA + 2 µM ssDNA reporter; 5. 0.6 µM crRNA + 5 µM ssDNA reporter; 6. 1 µM crRNA + 5 µM ssDNA reporter; 7. 2 µM crRNA + 5 µM ssDNA reporter; 8. 5 µM crRNA + 5 µM ssDNA reporter; 9. 1 µM crRNA + 10 µM ssDNA reporter; 10. 2 µM crRNA + 10 µM ssDNA reporter; 11. 5 µM crRNA + 10 µM ssDNA reporter; 12. 10 µM crRNA + 10 µM ssDNA reporter. Blue dots represent experimental groups with fluorescence signals below the threshold, red dots represent experimental groups with fluorescence signals above the threshold (positive signal), and open circles represent the negative control (N). Scatter plots and error bars indicate the mean ± standard deviation of three replicates. The red dashed line denotes the threshold. (**A**) Fluorescence detection results obtained using a multimode microplate reader (λex: 485 nm, λem: 520 nm). (**B**) Green fluorescence images captured under a 470 nm blue LED transilluminator. Red dashed circle in (**A**) and red dashed box in (**B**) indicate the optimal concentration combination (group 12: 10 µM crRNA + 10 µM ssDNA reporter). The combination of 10 µM crRNA and 10 µM ssDNA reporter (group 12) yielded the strongest visible green fluorescence, and the quantitative fluorescence reading was significantly higher than that of all other combinations, thus identifying this as the optimal concentration. Different letters (a–f) denote significant differences among groups (*p* < 0.05). Data represent the mean ± SD of three replicates (*n* = 3).

To determine the optimal reaction conditions for the RPA-CRISPR/Cas12a detection system, different concentrations of crRNA and ssDNA reporter probe were optimized. Among the 12 concentration combinations tested, the combination of 2 μM crRNA and 10 μM ssDNA reporter probe produced the brightest visible green fluorescence under the blue light LED transilluminator, and the quantitative reading from the microplate reader was also significantly higher than other combinations (Figure 3A), so this was determined as the optimal concentration combination.

**Figure 4:**
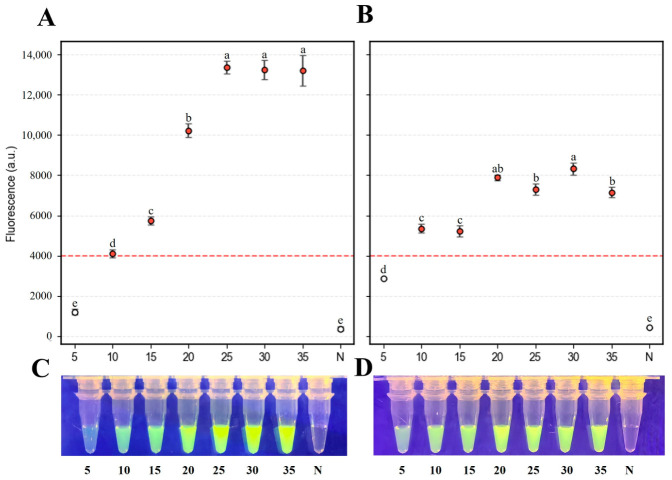
Optimization of RPA reaction time and Cas12a cleavage time in the RPA-CRISPR/Cas12a detection method. (**A**,**C**) Optimization of RPA reaction time: Scatter plots represent the mean fluorescence values ± standard deviation (SD) of three replicates at different reaction times (5, 10, 15, 20, 25, 30, 35, 40 min); N: negative control (ddH_2_O). (**B**,**D**) Optimization of Cas12a cleavage time: Scatter plots represent mean fluorescence values ± standard deviation of three replicates at different cleavage times (5, 10, 15, 20, 25, 30, 35, 40 min). (**A**,**B**) Detection results using a multimode microplate reader (λex = 485 nm, λem = 520 nm). (**C**,**D**) Green fluorescence images captured under a 470 nm blue light LED transilluminator. The red dashed line indicates the threshold (4000 a.u.). Red dots represent experimental groups with fluorescence signals above the threshold (positive signal), while open circles represent experimental groups with fluorescence signals below the threshold or the negative control (N). Different lowercase letters (a–e) indicate significant differences between groups (*p* < 0.05). Data represent the mean ± SD of three replicates (*n* = 3).

Under this concentration combination, the optimal reaction time for RPA amplification was further investigated. Seven time gradients from 5 to 35 min were set, along with a negative control, with 4000 a.u. as the positive threshold (approximately 10 times the fluorescence value of the negative control). The scatter plot and error bars showed that the fluorescence signal gradually increased with time (Figure 4A,C). At 5 min of RPA reaction, the fluorescence signal was discernible but not yet stably above the threshold line; at 10 min of reaction, the fluorescence signal stably exceeded 4000 a.u., reaching a reliable interpretation standard; extending the reaction time further increased the fluorescence intensity, but the increase slowed. Therefore, the optimal RPA reaction time was set to 10 min. Subsequently, using the 10 min RPA product as a template, the appropriate duration for the Cas12a cleavage reaction was evaluated. In tests with the same seven time gradients, 10 min of Cas12a cleavage yielded a stable and strong fluorescence signal, and extending the cleavage time did not significantly increase the fluorescence intensity (Figure 4B,D). Therefore, the optimal Cas12a cleavage time was determined to be 10 min. In summary, the entire amplification and detection process could be completed within 20 min (RPA 10 min + Cas12a 10 min), and a clear, discernible green fluorescence result could be obtained directly under a blue light LED transilluminator.

### 2.3. Specificity of the RPA-CRISPR/Cas12a Detection Method for Rapid Detection of Fusarium proliferatum

**Figure 5:**
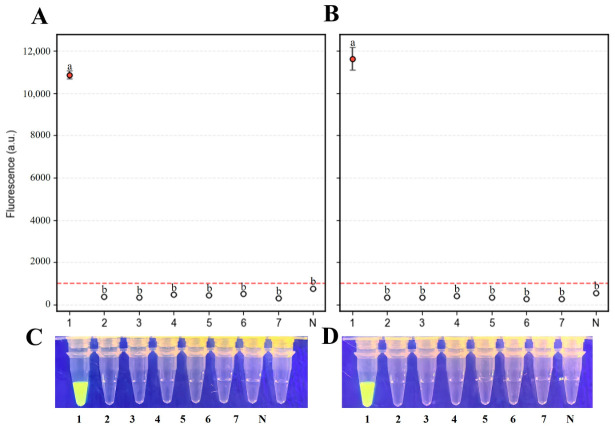
Specificity evaluation of the RPA-CRISPR/Cas12a detection method. (**A**) Intra-genus specificity verification: 1: *F. proliferatum* (Fpr1); 2: *F. fujikuroi* (Ffu1); 3: *F. graminearum* (Fgr1); 4: *F. circinatum* (A045-1); 5: *F. oxysporum* (Fox2); 6: *F. asiaticum* (Fas1); 7: *F. verticillioides* (Fve1); N: negative control (ddH_2_O). (**B**) Cross-genus/cross-kingdom specificity verification: 1: *F. proliferatum*; 2: *Bursaphelenchus xylophilus*; 3: *Pythium dissotocum*; 4: *P. spinosum*; 5: *F. fujikuroi*; 6: *P. plurisporium*; 7: *P. aphanidermatum*; N: negative control (ddH_2_O). (**C**,**D**) are the visible green fluorescence images of (**A**,**B**), respectively, under a 470 nm blue-light LED transilluminator. Scatter plots represent the mean fluorescence values ± standard deviation of three replicates. The red dashed line indicates the threshold (1000 a.u.). Red dots represent groups with fluorescence signals above the threshold (positive signal), while open circles represent groups with fluorescence signals below the threshold or the negative control (N). Different letters (a,b) indicate significant differences between groups (*p* < 0.05). Data represent the mean ± SD of three replicates (*n* = 3).

Primers *Fpro15953*-RPA-F1 and *Fpro15953*-RPA-R1 showed amplification specificity for *F. proliferatum*, producing an RPA product of the expected size only in the genomic DNA of *F. proliferatum*. Further verification was performed using the RPA-CRISPR/Cas12a assay, and the results are shown in Figure 5. In terms of intra-genus specificity, the fluorescence signal of *F. proliferatum* was well above the threshold line of 1000 a.u., while the fluorescence signals of closely related species such as *F. fujikuroi*, *F. graminearum*, *F. circinatum*, *F. oxysporum*, *F. asiaticum*, and *F. verticillioides* were all below the threshold line (Figure 4A). In cross-genus/cross-kingdom validation, *Bursaphelenchus xylophilus*, various *Pythium* species (*P. dissotocum*, *P. spinosum*, *P. plurisporium*, *P. aphanidermatum*), and the reference *F. fujikuroi* also produced no detectable fluorescence signal, remaining at the same background level as the negative control (Figure 5B). The three replicates of the above specificity tests were completely consistent, indicating that this method has good specificity for *F. proliferatum*.

### 2.4. Sensitivity of the RPA-CRISPR/Cas12a Detection Method for the Rapid Detection of Fusarium proliferatum

**Figure 6:**
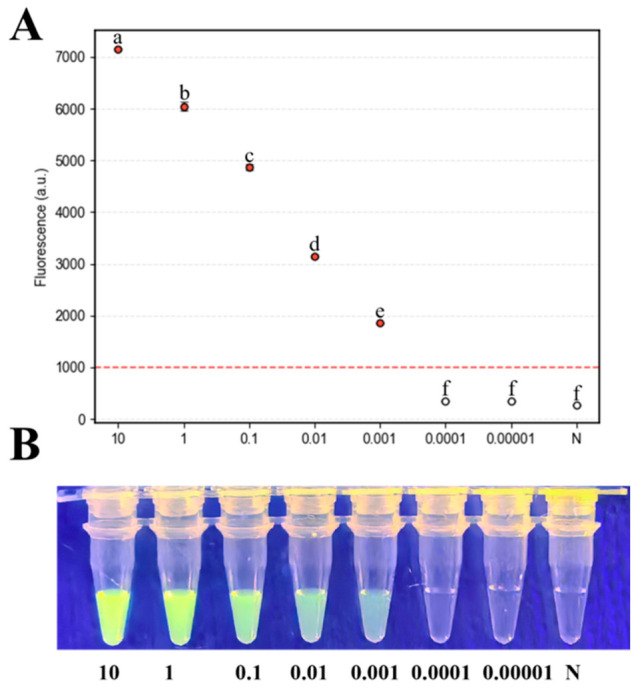
Sensitivity of the RPA-CRISPR/Cas12a method for the rapid detection of *Fusarium proliferatum*. (**A**) Fluorescence detection results using a multimode microplate reader (λex = 485 nm, λem = 520 nm); (**B**) Visible green fluorescence images under a 470 nm blue light LED transilluminator. Scatter plots represent mean fluorescence values ± standard deviation of three replicates for each concentration gradient. 1–8 are: 10 ng·μL^−1^, 1 ng·μL^−1^, 0.1 ng·μL^−1^, 0.01 ng·μL^−1^, 0.001 ng·μL^−1^, 0.0001 ng·μL^−1^, 0.00001 ng·μL^−1^, and negative control (N, ddH_2_O). The red dashed line indicates the threshold (1000 a.u.). Red dots represent concentrations with fluorescence signals above the threshold (positive signal), while open circles represent concentrations with fluorescence signals below the threshold or the negative control (N). Different lowercase letters (a–f) indicate significant differences between groups (*p* < 0.05). Data represent mean ± SD of three replicates (*n* = 3).

To evaluate the sensitivity of the RPA-CRISPR/Cas12a method for detecting *F. proliferatum*, the gDNA of *F. proliferatum* was serially diluted 10-fold to obtain seven concentration gradients from 10 ng·μL^−1^ to 0.00001 ng·μL^−1^, and 2 μL of each was used as a template. The results are shown in Figure 6. At gDNA concentrations of 10 ng·μL^−1^, 1 ng·μL^−1^, 0.1 ng·μL^−1^, and 0.01 ng·μL^−1^, the fluorescence signals stably exceeded the threshold line of 1000 a.u., and the reaction tubes showed clearly visible green fluorescence under the blue light LED transilluminator. At concentrations of 0.001 ng·μL^−1^, 0.0001 ng·μL^−1^, and 0.00001 ng·μL^−1^, the fluorescence signals dropped sharply below the threshold line, remaining at the same background level as the negative control (N), and were indistinguishable to the naked eye. Three replicate experiments yielded consistent results, thus determining the lower limit of detection of this method for *F. proliferatum* gDNA to be 0.01 ng·μL^−1^ (i.e., 10 pg·μL^−1^). Each concentration gradient was tested in three independent replicates under the same experimental conditions.

### 2.5. Detection Results of Fusarium proliferatum in Artificially Inoculated Cedrus deodara and Populus × hopeiensis Using RPA-CRISPR/Cas12a 

**Figure 7:**
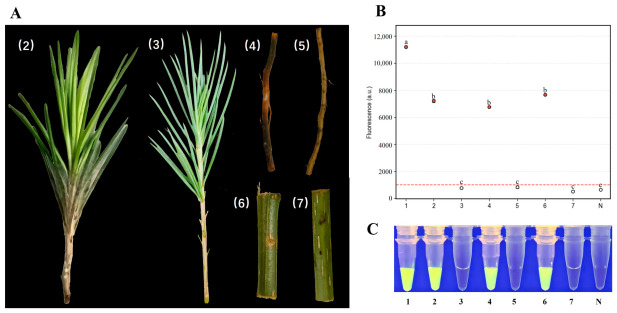
Detection of Fusarium proliferatum in artificially inoculated *Cedrus deodara* and *Populus × hopeiensis* samples using the RPA-CRISPR/Cas12a method. (**A**) Detection results of the inoculated and non-inoculated samples (groups 2–7): 2: *Cedrus deodara* needle sample artificially inoculated with *F. proliferatum*; 3: Non-inoculated *Cedrus deodara* needle sample (negative control); 4: Inoculated *Populus × hopeiensis* root segment sample; 5: Non-inoculated *Populus × hopeiensis* root segment sample (negative control); 6: Inoculated *Populus × hopeiensis* stem segment sample; 7: Non-inoculated *Populus × hopeiensis* stem segment sample (negative control). (**B**) Fluorescence intensity measured by a multimode microplate reader (λex = 485 nm, λem = 520 nm), including the positive control (1) and blank control (N); (**C**) Green fluorescence images captured under a 470 nm blue light LED transilluminator, also including groups 1 and N. Scatter plots represent mean fluorescence values ± standard deviation of three replicates. The red dashed line indicates the threshold (1000 a.u.). Red dots represent groups with fluorescence signals above the threshold (positive signal), while open circles represent groups with fluorescence signals below the threshold or the negative/blank control (N). (**B**) Fluorescence intensity measured by a multimode microplate reader (λex = 485 nm, λem = 520 nm). (**C**) Green fluorescence images captured under a 470 nm blue light LED transilluminator. Different lowercase letters (a–c) indicate significant differences between groups (*p* < 0.05).

Combining the EZ-D rapid extraction method with the RPA-CRISPR/Cas12a detection technology, we tested samples of *Cedrus deodara* needles, *Populus × hopeiensis* root segments, and *Populus × hopeiensis* stem segments artificially inoculated with *F. proliferatum*. The results are shown in Figure 7. The fluorescence signals of the positive control (pure culture gDNA of *F. proliferatum*) and the three artificially inoculated samples (*Cedrus deodara* needles, *Populus × hopeiensis* roots, *Populus × hopeiensis* stems) were all well above the threshold line, and the reaction tubes showed clearly visible green fluorescence under the blue light LED transilluminator. The non-inoculated *Cedrus deodara* needles, *Populus × hopeiensis* root segments, *Populus × hopeiensis* stem segments, and blank control produced no detectable fluorescence signal, remaining at the same background level as the negative control. The visible fluorescence results for all samples were quantitatively verified using a multimode microplate reader, and both were completely consistent (Figure 7A,B). These results indicate that the RPA-CRISPR/Cas12a detection method, combined with the EZ-D rapid extraction technology, can be effectively used for the rapid detection of *F. proliferatum* in different host plant tissues.

To further examine the distribution of *F. proliferatum* in infected plant tissues, we also tested adjacent asymptomatic areas (approximately 0.5–1 cm from the lesion edge) using the same RPA-CRISPR/Cas12a assay. As shown in Figure S2, all lesion-margin samples from *C. deodara* needles, *P.* × *hopeiensis* root segments, and *P.* × *hopeiensis* stem segments (groups 1, 4, 7) produced strong fluorescence signals above the threshold, whereas all adjacent asymptomatic tissues (groups 2, 5, 8), non-inoculated controls (groups 3, 6, 9), and blank controls (N) remained negative, with fluorescence values comparable to the background level. These results indicate that *F. proliferatum* DNA was predominantly localized within the necrotic lesion regions, while the pathogen load in nearby asymptomatic tissues was below the detection limit of the assay. Data represent the mean ± SD of three replicates (*n* = 3).

## 3. Discussion

*Fusarium proliferatum* is a filamentous fungal pathogen with an exceptionally broad host range, affecting both forest trees (e.g., *Cedrus deodara* and *Populus* spp.) and major cereal crops (maize, rice, wheat, sorghum) as well as cash crops like banana and onion [2,5]. It causes significant economic losses, and importantly, produces mycotoxins such as fumonisins that pose a threat to human and animal health via the food chain [31,32,33,34,35].

Traditional identification relies on pure culture and morphological observation of macro- and microconidia, but *Fusarium* species exhibit highly similar and overlapping traits, making this approach time-consuming (3–7 days) and expertise-dependent [2]. PCR-based methods have improved specificity and sensitivity [36,37], yet they still require thermal cyclers and gel electrophoresis, with a total time of 2.5–3 h, which limits their use for field diagnosis.

To simplify detection and reduce equipment dependence, isothermal amplification techniques, especially loop-mediated isothermal amplification (LAMP), have been developed [14,16,38]. LAMP-based assays have been applied to detect *F. proliferatum* in various crop diseases [39,40,41], but LAMP suffers from complex primer design and strict target-sequence requirements [14].

To overcome these technical obstacles, the present study successfully established and evaluated an RPA-CRISPR/Cas12a detection method targeting the *F. proliferatum*-specific gene *Fpro_15953*. To the best of our knowledge, this is the first time this methodological system has been applied for the rapid identification of *F. proliferatum* [30]. The *F. proliferatum* isolate (Fpr1) used in this study was verified by Koch’s postulates to be pathogenic to *C. deodara* needles, consistent with our previous characterization of this pathogen on *C. deodara* [42], ensuring consistency between the detection target and the disease diagnostic objective. In terms of assay design, RPA primers were first used to isothermally amplify the target gene (37 °C, 10 min), followed by secondary recognition of the amplicon by the Cas12a-crRNA complex and subsequent cleavage of the ssDNA reporter probe; the entire reaction process can be completed in approximately 20 min. Compared with other recently reported *Fusarium* detection technologies, this method offers a significant advantage in time efficiency (much shorter than the 73-minute RPA-Cas12a-LFD system) [30], while the dual-recognition mechanism effectively suppresses primer-dimer and non-specific amplification signals that may arise when RPA is used alone [17,19,38].

Specificity evaluation demonstrated that the assay produced a strong specific fluorescence signal only for *F. proliferatum* (Fpr1), whereas no positive reaction was observed for any of the 37 non-target isolates, including ten other *Fusarium* species such as *F. circinatum*, *F. fujikuroi*, *F. graminearum*, and *F. oxysporum*, ten other fungi including *Colletotrichum*, *Alternaria*, and *Botrytis* species, as well as eleven *Phytophthora* species, four other oomycetes, and two *Bursaphelenchus* nematodes. This high specificity is attributable to the synergistic effect of the two-step recognition mechanism: amplification of the *Fpro_15953* target region by RPA primers constitutes the first screening step, and the precise recognition of the amplicon by the Cas12a-crRNA complex followed by non-specific cleavage provides a second layer of assurance [19,20]. This cascade recognition mode greatly reduces the risk of cross-reaction with closely related species within the genus *Fusarium*.

In sensitivity validation, using 10-fold serial dilutions of purified *F. proliferatum* gDNA as a template, the limit of detection of the RPA-CRISPR/Cas12a method reached 0.001 ng·μL^−1^, which is comparable to some recently reported LAMP methods [38,40,41] but approximately two orders of magnitude more sensitive than conventional PCR (which requires approximately 0.1 ng·μL^−1^). Furthermore, this study examined the impact of the EZ-D rapid extraction method on detection stability. Detection results from naturally infected *C. deodara* needle samples collected in the field indicated that even when the EZ-D crude extract was used directly as the reaction template without further purification, the system could still achieve effective amplification and accurately discriminate the target pathogen [43]. This feature lowers the operational threshold for sample pre-treatment in the field and reduces the rigid dependence of traditional molecular diagnostics on high-quality DNA templates, thereby facilitating disease surveillance under basic conditions.

In terms of practical application, the RPA-CRISPR/Cas12a detection method exhibits several potential advantages with respect to operating conditions, result interpretation, and anti-interference capability. Both RPA amplification and Cas12a cleavage are performed at a constant temperature of 37 °C, requiring only a USB-powered incubator or a simple metal block heater to meet temperature requirements, thus avoiding dependence on precise PCR thermocyclers [15,18]. For result readout, the method allows for direct visual observation of the presence or absence of green fluorescence using a portable blue light LED transilluminator, without the need for gel electrophoresis or subsequent processing, making it particularly suitable for use at basic-level plant protection stations or quarantine checkpoints with limited equipment [22,44,45]. Moreover, the template obtained by the EZ-D rapid extraction method is sufficient to meet detection requirements, further shortening the time from sampling to result.

We also tested asymptomatic tissues: in inoculated samples, detection was positive only in lesion margins, not in adjacent asymptomatic areas (0.5–1 cm away), indicating that pathogen DNA is concentrated in necrotic zones. Therefore, while the assay is analytically sensitive, early diagnosis depends on sampling strategies targeting potential infection sites (e.g., wounds or natural openings) [46,47].

While the assay has clear advantages, several limitations should be addressed. Current sensitivity and stability data mainly come from pure cultures; field samples may contain inhibitors and low target abundance, requiring validation with more blind field samples [48]. The crRNA and primer designs in this study were based on the *Fpro_15953* gene sequence available in public databases; the conservation of this gene across *F. proliferatum* isolates from a broader range of geographical origins and genetic lineages has not yet been fully covered [11]. The conservation of *Fpro_15953* across diverse isolates and the limited availability of genomic sequences (only one isolate Fpr1 was used, and BLAST (NCBI BLAST+ version 2.17.0, performed on 23 July 2026) returned only the query) suggest that broader validation is needed [49,50]. However, the high specificity against 37 non-target isolates supports its utility as a marker [50]. Future studies should include more isolates from different origins and hosts to confirm conservation. Regarding specificity, certain *C. deodara* foliar pathogens (e.g., *Pestalotiopsis* and *Neofusicoccum* [51]) have not been tested and should be examined [9]. Cold-chain dependence of reagents limits deployment in remote areas; lyophilization could enable room-temperature storage [52]. Expanding to multiplex detection [53] could broaden field application. Finally, the biological function of *Fpro_15953* requires further study via gene knockout and overexpression.

## 4. Materials and Methods

### 4.1. Isolate Preservation and DNA Extraction

One *Fusarium proliferatum* isolate was recovered from symptomatic needles and branches of naturally diseased *Cedrus deodara* trees in Jiangsu, China, in 2022. The test isolates used in this study included: 1 isolate of *F. proliferatum* (Fpr1), 2 isolates of *F. circinatum*, 8 isolates of other *Fusarium* species (*F. acuminatum*, *F. asiaticum*, *F. fujikuroi*, *F. graminearum*, *F. oxysporum* Fox2, *F. oxysporum* Fox3, *F. solani*, *F. verticillioides*), 3 isolates of *Colletotrichum* species, 7 isolates of other fungi, 11 isolates of *Phytophthora* species, 4 isolates of other oomycetes, and 2 isolates of *Bursaphelenchus* nematodes, totaling 38 isolates. All isolates were obtained from the culture collection of the Department of Plant Pathology, Nanjing Forestry University (Nanjing, China). *F. proliferatum* and other fungal isolates were cultured on PDA medium in 90 mm Petri dishes at 25 °C in the dark for 3–5 days, and mycelium was scraped from the medium surface. Oomycetes were grown on V8 juice agar at 20 °C in the dark [54]. *Bursaphelenchus* nematodes were reared on mycelial mats of *Botrytis cinerea* grown on PDA at 25 °C in the dark for 5–7 days and were harvested using the Baermann funnel technique or collected directly from the culture plates [55]. Genomic DNA (gDNA) of all isolates was extracted using the DNAsecure Plant Genomic DNA Extraction Kit (Tiangen Biotech, Beijing, China) and quantified with a Nanodrop ND-1000 spectrophotometer (NanoDrop Technologies, Wilmington, DE, USA). All gDNA samples were stored at −20 °C for later use.

### 4.2. EZ-D Rapid Plant DNA Extraction

To identify a DNA extraction buffer suitable for rapid field detection, the EZ-D method was used with slight modifications [44]. Eight extraction buffers (A–H; formulations are listed in Table 1) were compared. A total of 100 mg of cedar or poplar tissue infected with Fusarium proliferatum was transferred into a 2 mL centrifuge tube containing 200 μL of nucleic acid extraction solution, and two YMZ-S grinding beads (Kairuida (Zhuhai, Guangdong, China) Technology Co., Ltd., Zhuhai, China) were added to the tube. The sample was vigorously shaken manually or by a vortex mixer for 10 s to fully disrupt fungal hyphae and plant tissues. The adsorption zone of an EZ-D cellulose filter strip was completely immersed in the resulting lysate and incubated for 10 s to capture nucleic acids from the samples. The filter strip was then transferred to a 1.5 mL centrifuge tube containing 100 μL of washing solution (10 mM Tris, pH 8.0, 0.1% Tween-20), with the adsorption zone fully submerged for 10 s to eliminate residual impurities that may interfere with subsequent nucleic acid amplification. Finally, the adsorption zone of the filter strip was immersed in a DNA amplification reaction solution or sterile water for 5–10 s to elute the bound nucleic acids. This simple three-step protocol allows for the rapid isolation of amplifiable template DNA, with the entire extraction procedure completed within 5 min. All Tris-based extraction solutions (A, B, D, E, and H) were adjusted to pH 8.0 with HCl or NaOH during preparation. For NaOH-based solutions F and G, no pH adjustment was performed; these solutions were prepared with their respective compositions as listed in Table 1 and used directly.

### 4.3. Design of RPA Primers, crRNA, and ssDNA Reporter

In this study, the *F. proliferatum*-specific gene *Fpro_15953* was selected as the detection target, and RPA primers and CRISPR RNA (crRNA) were designed against its sequence. RPA primers were designed using Primer Premier 6.0 software (Premier Biosoft, Palo Alto, CA, USA) following the recommendations of the RPA amplification kit manual [15,18]. The final forward primer was RPA-Fpro15953-F1 (5′-ATGAGCCTCATCATCATTGCCGAGTACACAAT-3′) and the reverse primer was RPA-Fpro15953-R1 (5′-TCCTGATGCTATAAAAAGGGAGGATACCCCAG-3′), generating an amplicon of 284 bp (Supplementary Figure S1). Meanwhile, the crRNA was designed using the CHOPCHOP web tool (http://chopchop.cbu.uib.no/) [56], with the sequence 5′-UAAUUUCUACUAAGUGUAGAUCCUCAUCGAGAUGGCUGUGU-3′. This crRNA does not overlap with the RPA primer sequences and targets a conserved region of the amplicon. The single-stranded DNA (ssDNA) reporter probe was labeled with a 6-FAM fluorophore at the 5′ end and a BHQ-1 quencher at the 3′ end (5′-6-FAM-TTATT-BHQ-1-3′). The crRNA and ssDNA reporter probe were synthesized by GenScript (Nanjing, China) and stored at −80 °C.

### 4.4. RPA-CRISPR/Cas12a Detection Method

The entire 20 min detection process consisted of two steps: RPA amplification (10 min) and CRISPR/Cas12a detection (10 min) (Figure 1). The RPA reaction was performed in a 50 μL system using an RPA amplification kit (Lesheng Biotechnology Co., Ltd., Wuxi, China) according to the manufacturer’s instructions [15,18]. Each reaction contained: 2 μL forward primer Fpro15953-RPA-F1 (10 μM), 2 μL reverse primer Fpro15953-RPA-R1 (10 μM), 25 μL rehydration buffer, 2 μL genomic DNA (10 ng·μL^−1^), and 16 μL double-distilled water, for a total volume of 47 μL. After brief centrifugation (4000 rpm, 5 s), 3 μL of activator (provided with the kit) was added to the cap of the reaction unit. The unit was closed, centrifuged again at 4000 rpm for 5 s, and manually shaken for 3 s to mix thoroughly. The reaction was carried out at 37 °C for 10 min. After the reaction, the tube was shaken, centrifuged at 4000 rpm for 5 s, and 2 μL of the RPA product was taken for downstream detection.

The CRISPR/Cas12a detection system (50 μL) contained 38 μL double-distilled water, 5 μL reaction buffer, 3 μL crRNA, 1 μL Cas12a protein, 1 μL ssDNA reporter probe, and 2 μL of the above RPA product [19,20]. The mixture was centrifuged at 4000 rpm for 5 s and incubated at 37 °C for 10 min. Results were interpreted in two ways: (1) fluorescence intensity measured using a multimode microplate reader (λex 485 nm, λem 520 nm); and (2) direct visual observation of green fluorescence under a 470 nm blue LED transilluminator. Negative controls showed no fluorescence signal or visible green fluorescence.

To optimize detection performance, the following twelve concentration combinations of crRNA and ssDNA reporter probe were screened: (1) 40 nM crRNA + 40 nM ssDNA reporter; (2) 80 nM crRNA + 500 nM ssDNA reporter; (3) 300 nM crRNA + 1.4 nM ssDNA reporter; (4) 0.5 µM crRNA + 2 µM ssDNA reporter; (5) 0.6 µM crRNA + 5 µM ssDNA reporter; (6) 1 µM crRNA + 5 µM ssDNA reporter; (7) 2 µM crRNA + 5 µM ssDNA reporter; (8) 5 µM crRNA + 5 µM ssDNA reporter; (9) 1 µM crRNA + 10 µM ssDNA reporter; (10) 2 µM crRNA + 10 µM ssDNA reporter; (11) 5 µM crRNA + 10 µM ssDNA reporter; (12) 10 µM crRNA + 10 µM ssDNA reporter. A negative control with ddH_2_O replacing the crRNA and ssDNA reporter probe was included for each combination. All reactions were performed at least three times. The standard deviation of three replicates was calculated using the STDEV.P function, and statistical analysis was performed using GraphPad Prism 8 software (GraphPad Software Inc., San Diego, CA, USA) with the Student’s *t*-test. A *p* < 0.05 was considered statistically significant.

### 4.5. Specificity and Sensitivity of the RPA-CRISPR/Cas12a Assay

The specificity of the assay was evaluated using all 38 isolates listed in Table 2 (including 1 isolate of *F. proliferatum*, 2 isolates of *F. circinatum*, 7 isolates of other *Fusarium* species, 3 isolates of *Colletotrichum* species, 6 isolates of other fungi, 10 isolates of *Phytophthora* species, 4 isolates of other oomycetes, and 2 isolates of *Bursaphelenchus* nematodes). Purified genomic DNA (100 ng) was used as a template. A positive control (100 ng gDNA of the representative *F. proliferatum* isolate) and a no-template control (NTC, double-distilled water) were included in each reaction set. These non-target isolates were selected based on three criteria: (i) phylogenetically closely related *Fusarium* species (*F. fujikuroi*, *F. verticillioides*, *F. graminearum*, *F. oxysporum*) to challenge the assay’s intra-genus specificity; (ii) other common pathogens that may co-infect the same hosts (*C. deodara* and *Populus* spp.), such as *Colletotrichum* spp., *Botryosphaeria dothidea*, and *Alternaria alternata*, to assess potential cross-reactions in complex host microenvironments; and (iii) taxonomically distant organisms including oomycetes (*Phytophthora* and *Pythium* spp.) and nematodes (*Bursaphelenchus* spp.), which often co-exist with *Fusarium* in soil, rhizosphere, or plant tissues and may cause cross-interference in field samples. Although not all test isolates were directly recovered from *C. deodara* or *Populus*, they represent the major biological groups that may co-occur with *F. proliferatum* in agricultural and forest ecosystems, thereby providing a rigorous evaluation of the assay’s specificity.

To determine the detection sensitivity, *F. proliferatum* gDNA was 10-fold serially diluted to concentrations ranging from 100 ng·μL^−1^ to 0.00001 ng·μL^−1^ (i.e., 10 ng·μL^−1^, 1 ng·μL^−1^, 0.1 ng·μL^−1^, 0.01 ng·μL^−1^, 0.001 ng·μL^−1^, 0.001 ng·μL^−1^, and 0.00001 ng·μL^−1^) and used as DNA template in the RPA-CRISPR/Cas12a assay. A negative control (NTC, ddH_2_O) was included in each reaction set. All specificity and sensitivity experiments were performed with at least three technical replicates (*n* = 3) per sample in each independent run. For key experiments, including sensitivity determination, the entire procedure was repeated in three independent biological replicates (*n* = 3), starting from fresh fungal cultures. Data are presented as the mean ± standard deviation (SD). The standard deviation was calculated using the STDEV.P function, and the Student’s *t*-test was performed using GraphPad Prism 8 to compare the significance of differences between experimental and control groups (*p* < 0.05).

### 4.6. RPA-CRISPR/Cas12a Assay for Detection of F. proliferatum in Inoculated Cedrus and Populus 

Healthy, detached young branches of *C. deodara* and stem and root segments of *Populus* × *hopeiensis* were collected and surface-sterilized sequentially with 75% ethanol for 1 min and 1% sodium hypochlorite for 30 s, rinsed three times with sterile water, and blotted dry with sterile filter paper. *F. proliferatum* was inoculated on PDA plates and cultured in the dark at 25 °C for 5 days. Uniform mycelial plugs (0.5 mm diameter) were taken from the colony margin using a cork borer and inoculated onto the surface of the above tissues by wound inoculation [56]; sterile agar plugs of the same size served as negative controls. The inoculated materials were placed in a growth chamber under conditions of 25 °C, a 12 h/12 h light/dark cycle, and 70% relative humidity. After 5–7 days of culture, *C. deodara* needles inoculated with *F. proliferatum* exhibited typical blight symptoms, with reddish-brown to dark brown necrotic lesions expanding from the inoculation sites. On *P.* × *hopeiensis* stem and root segments, the infection started at the inoculation points and gradually spread outward, with infected tissues becoming soft and discolored. In contrast, the mock-inoculated (sterile agar plug) control groups remained symptomless throughout the experiment. Approximately 1 g of tissue from the margin between diseased and healthy tissue was sampled from each treatment group and thoroughly ground using a grinder (XB-M101, Lingrui, Shanghai, China). DNA was extracted from 0.1 g of the ground sample using the optimized EZ-D method described in Section 4.2 [43]: 200 μL of TPS extraction buffer (100 mM Tris [pH 8.0], 1 M NaCl, 10 mM EDTA) was added, the tissue was homogenized with a disposable pestle, and the homogenate was left to stand at room temperature for 5 min; then 200 μL of phenol:chloroform:isoamyl alcohol (25:24:1) was added, vortexed vigorously, and centrifuged at 12,000 rpm for 2 min. The aqueous supernatant was directly used as the DNA template for the RPA-CRISPR/Cas12a assay. In each replicate experiment, purified *F. proliferatum* gDNA (10 ng·μL^−1^) and ddH_2_O were used as positive and negative controls (N), respectively. Three independent replicates were performed for each treatment. The standard deviation was calculated, and the Student’s *t*-test was performed using GraphPad Prism 8 to compare differences between experimental and control groups (*p* < 0.05). All experiments were performed with at least three technical replicates (*n* = 3). For each treatment group, the entire procedure was repeated in three independent biological replicates (*n* = 3). Data are presented as the mean ± standard deviation (SD).

## 5. Conclusions

This study established an RPA-CRISPR/Cas12a rapid detection system targeting the *Fusarium proliferatum*-specific gene *Fpro_15953*, which combines high specificity, sensitivity, and operational convenience. This method overcomes the shortcomings of traditional morphological identification, which is time-consuming and experience-dependent, and eliminates the reliance on thermocyclers required by PCR. In this system, both RPA amplification and Cas12a cleavage are shortened to 10 min each, with a total detection time of only about 20 min (still ≤30 min even including crude DNA extraction), making it more suitable for rapid field screening scenarios. The method has been successfully used to detect artificially infected *Cedrus deodara* and *Populus × hopeiensis* samples, verifying its feasibility for application at the grassroots level. It can not only serve as an early warning of forest diseases caused by *F. proliferatum*, but also provide a new tool for the rapid diagnosis of cereal crop diseases such as rice panicle rot and maize ear rot. This study lays a foundation for the development of a portable fluorescence detection terminal, with good application prospects and room for optimization and expansion.

## Figures and Tables

**Table 2:** Fungal, oomycete, and nematode isolates used in this study for specificity testing of the RPA-CRISPR/Cas12a assay.

No.	Scientific Name	Isolate	Host Plant	Source	Detection Result
1	*Fusarium proliferatum*	Fpr1	*Cedrus sp.*	Jiangsu, China	√
2	*F. circinatum*	A045-1	*Pinus* sp.	Jiangsu, China	×
3	*F. circinatum*	A045-3	*Pinus* sp.	Shanghai, China	×
4	*F. acuminatum*	Fac1	*Rhizophora apiculata*	Guangdong, China	×
5	*F. asiaticum*	Fas1	*Triticum aestivum*	Jiangsu, China	×
6	*F. fujikuroi*	Ffu1	*Oryza sativa*	Jiangsu, China	×
7	*F. graminearum*	Fgr1	*Triticum aestivum*	Jiangsu, China	×
8	*F. oxysporum*	Fox2	*Pinus* sp.	Jiangsu, China	×
9	*F. oxysporum*	Fox3	*Pinus* sp.	Jiangsu, China	×
10	*F. solani*	Fso1	*Pinus* sp.	Jiangsu, China	×
11	*F. verticillioides*	Fve1	*Oryza sativa*	Jiangsu, China	×
12	*Colletotrichum gloeosporioides*	SMCG1	*Cunninghamia lanceolata*	Jiangsu, China	×
13	*C. siamense*	YK102	*Cunninghamia lanceolata*	Jiangsu, China	×
14	*C. graminicola*	YB7	*Cunninghamia lanceolata*	Jiangsu, China	×
15	*Alternaria alternata*	CTAT8	*Cucumis sativus*	Heilongjiang, China	×
16	*Botryosphaeria dothidea*	JGT01	*Aucuba japonica*	Jiangxi, China	×
17	*Verticillium dahliae*	Vda1	*Gossypium* sp.	Jiangsu, China	×
18	*Rhizoctonia solani*	Rso1	*Arachis hypogaea*	Jiangsu, China	×
19	*Magnaporthe grisea*	Guy11	*Oryza sativa*	Japan	×
20	*Botrytis cinerea*	Bci1	*Cucumis sativus*	Jiangsu, China	×
21	*Aspergillus flavus*	NJC03	*Actinidia chinensis*	Shanxi, China	×
22	*Phytophthora cinnamomi*	ATCC15400	*Pinus* sp.	Jiangsu, China	×
23	*P. hibernalis*	CBS 270.31	*Citrus sinensis*	USA	×
24	*P. syringae*	CBS 132.23	*Citrus reticulata*	UK	×
25	*P. infestans*	Pin1	*Solanum tuberosum*	Fujian, China	×
26	*P. sojae*	R20	*Glycine max*	Jiangsu, China	×
27	*P. ramorum*	EU1 2275	*Quercus palustris*	UK	×
28	*P. nicotianae*	Pn-DC7	*Nicotiana tabacum*	Jiangsu, China	×
29	*P. cactorum*	Pcac1	*Malus pumila*	Fujian, China	×
30	*P. capsici*	Pcap1	*Capsicum annuum*	Jiangsu, China	×
31	*P. pini*	Ppini	*Rhododendron pulchrum*	Jiangsu, China	×
32	*P. palmivora*	Ppa1	Iridaceae spp.	Yunnan, China	×
33	*Phytopythium littorale*	Phlitro	*Rhododendron pulchrum*	Jiangsu, China	×
34	*P. helicoides*	Phe1	*Rhododendron pulchrum*	Jiangsu, China	×
35	*Pythium aphanidermatum*	SDHL-1	*Head lettuce*	Shandong, China	×
36	*P. ultimum*	PU-1	*Oryza sativa*	Guangdong, China	×
37	*Bursaphelenchus xylophilus*	JS-43	*Pinus* sp.	Jiangsu, China	×
38	*B. mucronatus*	Bmucro	*Pinus* sp.	Jiangsu, China	×

√, positive; ×, negative.

## Data Availability

The raw data supporting the conclusions of this article will be made available by the authors on request.

## Supplementary Materials

The following supporting information can be downloaded at: https://www.mdpi.com/article/10.3390/plants15152352/s1, Figure S1. Schematic diagram showing the positions of RPA primers and crRNA on the target gene*Fpro_15953*. Figure S2. Detection of *F. proliferatum* in lesion margin and adjacent asymptomatic tissues of artificially inoculated *Cedrus deodara* and *Populus* × *hopeiensis* samples using the RPA-CRISPR/Cas12a assay. Green fluorescence images captured under a 470 nm blue light LED transilluminator. Groups: 1, Positive control (PTC, purified *F. proliferatum* DNA); 2, *C. deodara* needle from lesion margin (inoculated); 3, *C. deodara* needle from adjacent asymptomatic tissue (~0.5–1 cm from lesion); 4, non-inoculated *C. deodara* needle; 5, *P.* × *hopeiensis* root from lesion margin (inoculated); 6, *P.* × *hopeiensis* root from adjacent asymptomatic tissue; 7, non-inoculated *P.* × *hopeiensis* root; 8, *P.* × *hopeiensis* stem from lesion margin (inoculated); 9, *P.* × *hopeiensis* stem from adjacent asymptomatic tissue; 10, non-inoculated *P.* × *hopeiensis* stem; N, blank control (no template). Positive signals (green fluorescence) were observed only in the positive control (group 1) and lesion-margin samples (groups 2, 5, 8), while adjacent asymptomatic tissues (groups 3, 6, 9), non-inoculated controls (groups 4, 7, 10), and the blank control (N) remained negative. Data represent the mean ± SD of three replicates (*n* = 3).
