# Supplementary material for: RPA-CRISPR/Cas12a-Mediated Isothermal Amplification Technology for Visual Detection of Fusarium proliferatum
Source: Plants (Basel). 2026 Jul 30;15(15):2352. doi: 10.3390/plants15152352 (PMC13467610; doi:10.3390/plants15152352)
Supplement: Supplementary file 1 [file plants-15-02352-s001.zip › plants-4380950-Supplementary file.pdf]

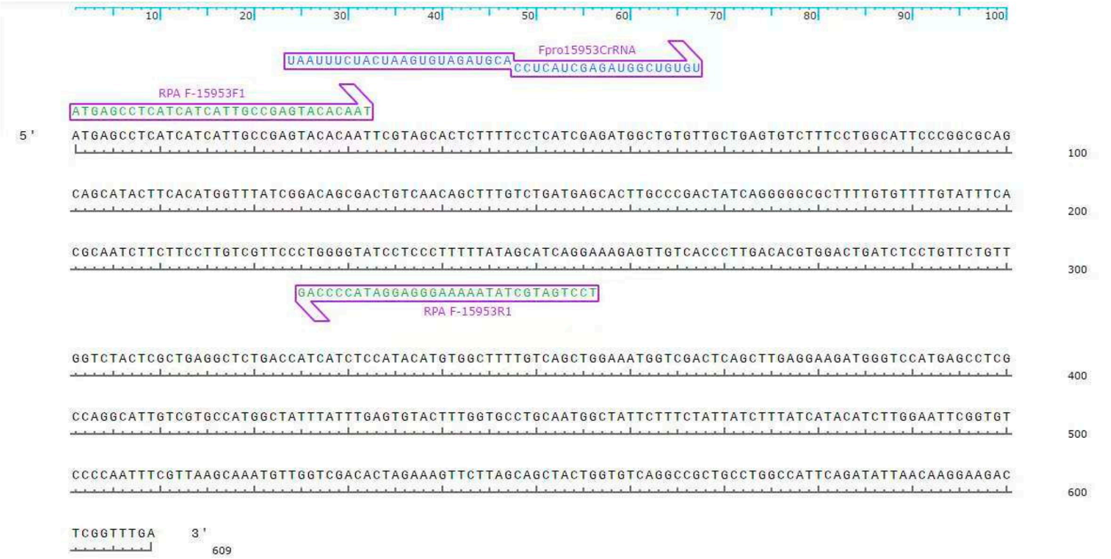


Figure S1. Schematic diagram showing the positions of RPA primers and crRNA on the target gene*Fpro_15953*.

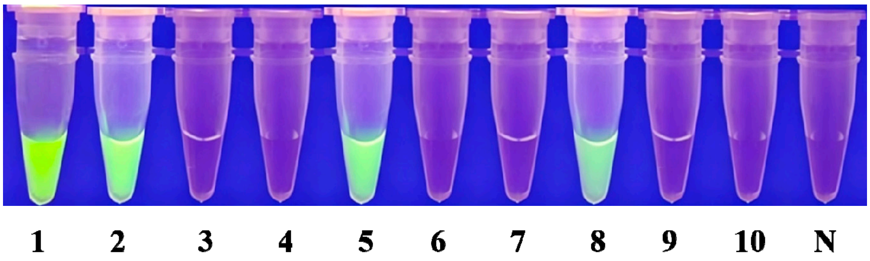


Figure S2. Detection of *F. proliferatum* in lesion margin and adjacent asymptomatic tissues of artificially inoculated *Cedrus deodara* and *Populus* × *hopeiensis* samples using the RPA-CRISPR/Cas12a assay. Green fluorescence images captured under a 470 nm blue light LED transilluminator. Groups: 1, Positive control (PTC, purified *F. proliferatum* DNA); 2, *C. deodara* needle from lesion margin (inoculated); 3, *C. deodara* needle from adjacent asymptomatic tissue (~0.5–1 cm from lesion); 4, non-inoculated *C. deodara* needle; 5, *P.* × *hopeiensis* root from lesion margin (inoculated); 6, *P.* × *hopeiensis* root from adjacent asymptomatic tissue; 7, non-inoculated *P.* × *hopeiensis* root; 8, *P.* × *hopeiensis* stem from lesion margin (inoculated); 9, *P.* × *hopeiensis* stem from adjacent asymptomatic tissue; 10, non-inoculated *P.* × *hopeiensis* stem; N, blank control (no template). Positive signals (green fluorescence) were observed only in the positive control (group 1) and lesion-margin samples (groups 2, 5, 8), while adjacent asymptomatic tissues (groups 3, 6, 9), non-inoculated controls (groups 4, 7, 10), and the blank control (N) remained negative. Data represent the mean ± SD of three replicates (*n* = 3).
